# Enhancing the reporting of implementation research

**DOI:** 10.1186/s13012-017-0546-3

**Published:** 2017-02-08

**Authors:** Paul M. Wilson, Anne Sales, Michel Wensing, Gregory A. Aarons, Signe Flottorp, Liz Glidewell, Alison Hutchinson, Justin Presseau, Anne Rogers, Nick Sevdalis, Janet Squires, Sharon Straus

**Affiliations:** 1 Alliance Manchester Business School, University of Manchester, Booth Street East, Manchester, M15 6PB UK; 2 Department of Veterans Affairs Center for Clinical Management Research, VA Ann Arbor Healthcare System, Ann Arbor, MI USA; 3 University of Michigan, Ann Arbor, MI USA; 4 University of Heidelberg, Heidelberg, Germany; 5 University of California, San Diego, CA USA; 6 Norwegian Knowledge Centre for the Health Services, Oslo, Norway; 7 University of Leeds, Leeds, UK; 8 Deakin University, Melbourne, Australia; 9 Ottawa Hospital Research Institute, Ottawa, Canada; 10 University of Southampton, Southampton, UK; 11 King’s College London, London, UK; 12 University of Ottawa, Ottawa, Canada; 13 University of Toronto, Toronto, Canada

## Abstract

In the 10 years since the inception of *Implementation Science*, we have witnessed a continued rise in the number of submissions received, reflecting the continued global interest in methods to enhance the uptake of research findings into healthcare practice and policy. We receive over 750 submissions annually, and there is now a large gap between what is submitted and what gets published. In this editorial, we restate the journal scope and current boundaries. We also identify some specific reporting issues that if addressed will help enhance the scientific reporting quality and transparency of the manuscripts we receive. We hope that this editorial acts as a further guide to researchers seeking to publish their work in *Implementation Science*.

## Background

**Fig. 1:**
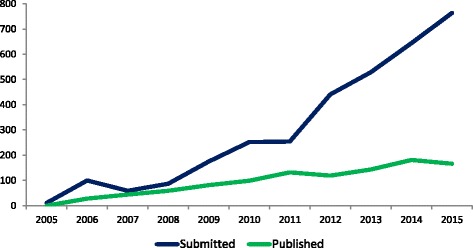
Manuscripts submitted to and accepted for publication in *Implementation Science*

In the 10 years since the inception of *Implementation Science*, we have witnessed a continued rise in the number of manuscripts submitted. We now receive over 750 submissions annually (see Fig. 1), reflecting the continued interest from researchers, funders and health professionals and policy makers in promoting the uptake of research findings into healthcare practice and policy. The number of manuscripts published in *Implementation Science* remains steady at around 150 per year.

The large gap between what is submitted and what gets published is driven by two key issues, namely scope and scientific quality. This editorial aims to address both of these issues and act as a further guide to researchers seeking to publish their work in *Implementation Science*.

### Scope and boundaries

In 2015, we reviewed and provided a detailed explanation and elaboration of our journal scope [1]. As of 2017, we have no plans to expand further the boundaries of our scope at this point in time. Therefore, our focus remains on the publication of studies examining the implementation of evidence-based healthcare interventions, practices or policies or the de-implementation of those demonstrated to be of low or no clinical benefit or even harmful.

For implementation effectiveness, we seek to publish studies that employ rigorous experimental or quasi-experimental designs regardless of whether they report effects or no effects. By rigorous, we mean those designs that would be eligible for inclusion in the Cochrane EPOC reviews [2]. This can include type 2 or type 3 hybrid designs where there is a dual a priori focus on assessing clinical effectiveness and implementation strategies, [3] but only where there is a clear justification and major element of implementation research. Type 2 designs have dual focus effectiveness and implementation outcomes, here, for example, testing both the effectiveness of brief cognitive behavioural therapy and the implementation strategies [4]. A type 3 is where the primary emphasis is on evaluating implementation, in this instance of a diabetes prevention programme, but where data on clinical outcomes are also collected [5].

We continue to receive a considerable number of studies testing novel clinical or population health interventions, where the effectiveness of the intervention or practice has yet to be established. As our scope focuses on the implementation of interventions of demonstrated effectiveness, we routinely reject these manuscripts (and offer transfer to other BMC journals). These exclusion criteria extend also cover type 1 hybrid designs where the focus is on testing effects of a clinical intervention on relevant outcomes whilst observing and gathering information on implementation [3]. For instance, a clinical trial of primary care management of survivors of sepsis focused on patients’ quality of life as the primary outcome also comprised a range of measures of implementation aspects [6]. Studies of this type fall outside of our journal scope.

Alongside effectiveness, the journal scope also includes economic evaluation and qualitative research that examines different aspect of interventions and context which contribute to effectiveness. This includes the study of adaptation and fidelity, mechanisms of impact and contextual influences on implementation and outcomes, sustainability and scalability as well as the study of influences on provider, patient and organisational behaviour. Crucially, we expect the methods employed in such studies to be an appropriate fit to the question(s) being addressed and be informed by relevant conceptual frameworks.

We also welcome articles that present new methods and articles that question or challenge existing implementation policies, practices, evidence or theory and suggest modifications or alternatives. However, it is worth noting that there is no shortage of frameworks and theories relevant to implementation research [7, 8]. So rather than adding to the current pot, our preference is for empirical studies that build and advance the existing theoretical base. With debate papers, we reject those that fail to ground the central argument within the existing implementation research literature. Many debate papers would be of greater relevance if the arguments posed were based upon systematic reviews of the relevant evidence.

Table 1 presents the types of manuscripts likely to be accepted by or rejected from *Implementation Science*. This should assist prospective authors to judge whether the journal is a suitable home for their research.

**Table 1:** Factors promoting the likelihood of acceptance or rejection from *Implementation Science* by manuscript type

Type of manuscript	Factors promoting likelihood of acceptance	Factors promoting likelihood of rejection	Preferred reporting methods
Debate	Papers which question or challenge existing implementation policies, practices, evidence or theory and suggest modifications or alternatives	Papers which fail to contextualise in the literature or demonstrate how they build upon the existing implementation research literature	N/A
Effectiveness	Studies that fit our journal scope and that employ rigorous experimental or quasi experimental designs (i.e. designs eligible for inclusion in Cochrane EPOC reviews) And Evaluate the implementation of an evidence-based practice or policy or de-implementation of those demonstrated to be of low or no clinical benefit	Studies which lack a rigorous study design such as quality improvement reports, service evaluations or uncontrolled before-after studies Studies evaluating the effectiveness of novel clinical, organisational, public health or policy interventions	CONSORT for Trials
Economic evaluation	Any cost effectiveness analysis that compares the costs and outcomes of two or more implementation strategies	Cost and cost consequences analysis where disaggregated costs and outcomes are presented	CHEERS
Intervention development reports	Prepared and submitted prior to the reporting of the effectiveness of the intervention Plans for (robust) evaluation are made explicit Providing empirical and/or theoretical rationale	Post hoc submission (submitted after the reporting of the effectiveness of the intervention) No plans for (robust) evaluation	
Methodology	Articles that present methods which may either be completely new or offer an improvement to an existing method Articles reporting empirical comparisons of one or more methodological approaches or which clearly state what they add to existing literature	Descriptive accounts of largely established methods without any associated novel methodological insights	N/A
Pilot and feasibility studies	Studies that fit our journal scope and conducted with the explicit purpose of assessing feasibility and planning for an intervention that is expected to contribute to existing knowledge Studies indicating how a subsequent study will draw from the pilot study Clear plans for further evaluation or where there are clear reasons for not	No justification for conduct Over claim on basis of results	
Process evaluation	Studies that fit our journal scope and are submitted contemporaneously with or following reports of intervention effectiveness and that take account of the main evaluation outcomes Studies evaluating fidelity of implementation, mechanisms of impact and or contextual influences on implementation and outcomes	Process evaluations submitted in advance of the conduct of the main effectiveness analysis (it cannot be clear if they are explaining an effect or the absence of an effect) Process evaluations that do not take account of the main evaluation outcomes	
Protocols	Protocols that fit our journal scope and inclusion criteria for rigorous study designs And That have been through a competitive peer review process to receive funding from a nationally or internationally recognised research agency And That have received appropriate ethics review board approval And That have been submitted within three possible time points: (1) Within 3 months of ethics approval, (2) Prior to enrolment of the first participant/cluster (3) Before the end of participant/cluster recruitment (i.e. prior to the commencement of data cleaning or analysis)	Protocols that have not been the subject of peer review by a national or international research agency Protocols that have received ethics review board approval Protocols for quality improvement or service evaluations, which lack a rigorous study design Protocols for pilot or feasibility studies Protocols for systematic reviews and other types of synthesis (we usually refer these to the BMC journal, systematic reviews) Protocols that are submitted for studies where data cleaning and analysis have begun	As SPIRIT is developed for clinical trials, we prefer authors to complete as far as they can the CONSORT checklist or appropriate extension
Qualitative studies	Studies that fit the journal scope and meet applicable criteria for quality and validity	Studies where there are doubts whether planned data saturation has been achieved Single site case studies with limited typicality Studies that fail to link to relevant theory or without contextualisation and with little reference to previous relevant qualitative studies or reviews	
Short reports	Brief reports of data from original research which present relatively modest advances in knowledge or methods	Reports of meetings, ‘doing implementation’ or ‘lessons learned’	N/A
Systematic reviews and other syntheses	Systematic reviews and other types of synthesis (such as rapid, realist or scoping) that fit our journal scope and which may cover issues such as the effects of implementation interventions and or influences on the uptake of evidence	Non-systematic or narrative literature reviews that fail to use explicit methods to identify, select, and critically appraise relevant research Reviews and syntheses that fail to adhere to recognised quality and reporting standards	PRISMA RAMESES for realist reviews

### Enhancing reporting

Alongside failure to meet scope requirements, poor scientific quality remains a common reason for rejection. Promoting the development, refinement and quality of implementation research was a key aim of the founding editors [9] and remains so today. We therefore continue to support and promote efforts to improve research quality and transparency.

### Prospective trial registration

*Implementation Science* supports initiatives to improve the reporting of randomised trials. We have adopted the ICMJE recommendation [10] and only normally consider for publication trials that have been registered with an appropriate publicly available trials database prior to enrolment of the first participant/cluster. We will consider retrospectively registered trials on a case by case basis but will require authors to explain the reason(s) for the delayed registration.

Whilst there are no fixed rules about the registration of other study designs, we strongly encourage authors of systematic reviews to prospectively register their review with PROSPERO or other publicly accessible registries.

### Enhancing research reporting

Over the last decade we have routinely required authors submitting manuscripts that report trials to complete the CONSORT checklist or relevant extension. Similarly, a requirement to complete the PRISMA checklist has been enforced for authors submitting systematic reviews. No other checklists have been routinely or uniformly enforced. As a journal that receives manuscripts covering a wide range of study designs, this has resulted in variation in the standards of reporting of the research that we publish.

Because our aim is to promote research quality and transparency, and as an aid to our readers, reviewers and editors, we now require authors submitting manuscripts (regardless of study design) to complete and include a design appropriate reporting checklist.

The website of the EQUATOR Network provides details of all available reporting guidelines (www.equator-network.org). Authors of manuscripts (regardless of study design) should refer to EQUATOR and ensure that they complete and include a design appropriate reporting checklist with their submission. Table 1 includes details of our preferred reporting formats; for those research types where consensus is lacking on reporting format (for example, in qualitative research), we encourage authors to select their preferred checklist.

Improving the quality of intervention description is as much an issue for implementation research as it is for other evaluations of complex interventions. Without sufficient detail, it is difficult for readers to determine what was actually implemented and/or for other researchers to use or replicate the intervention in other studies. Whilst TIDieR has been proposed for use in conjunction with the CONSORT guidelines for trials, [11] improved intervention description is relevant across all evaluative study designs. Other relevant standards for reporting implementation interventions (Standards for Reporting Implementation studies —StaRI) [12] and for reporting behaviour change interventions (Workgroup for Intervention Development and Evaluation Research—WIDER) [13] have been developed and are available. We encourage authors to select their preferred guideline to enhance reporting of interventions.

With all submissions, we expect authors to clearly articulate what is already known and what their work adds to existing knowledge, theory and thinking in the field. Many submissions currently fail to set the work in the context of the existing literature. And so we will continue to reject manuscripts that do not clearly build on current knowledge and understanding or appear to provide limited contributions.

### Open Science

As an open access journal (with open peer review), we are committed to making research and the datasets upon which it is based, publicly accessible. A number of different data sharing approaches have now been adopted across the health and medical literature [14]. At *Implementation Science*, we have adopted the policies on data availability of our publisher BMC. As part of online article submission, we now ask authors to include an “Availability of Data and Materials” section in their manuscript detailing the conditions by which the data supporting their findings can be accessed. Authors who do not wish to share their data must include a formal statement that data will not be shared and give the reason why. Full details of BMC policies can be found under the Instructions for Authors section of our website.

## Conclusion

In this editorial, we have identified some specific reporting issues that if addressed will help enhance the scientific reporting quality and transparency of the manuscripts we receive. We also encourage prospective authors to familiarise themselves with the journal scope and boundaries before making a submission. We look forward to the next 10 years as the field continues to grow and evolve and to receiving research that continues to enhance the uptake of evidence-based practices or policies to improve the quality and delivery of healthcare.
